# SOLUBLE INTERCELLULAR ADHESION MOLECULE (SICAM-1) AS A BIOMARKER OF VASCULAR COGNITIVE IMPAIRMENT IN OLDER ADULTS

**DOI:** 10.1093/geroni/igz038.959

**Published:** 2019-11-08

**Authors:** Rosalinda Sanchez Arenas, Michael A Gregory, Leticia Manuel-Apolinar

**Affiliations:** 1 IMSS, Mexico, Mexico City, Mexico; 2 McMaster University, Hamilton, Ontario, Canada

## Abstract

Background: Endothelial dysfunction and subsequent inflammation contribute to the development of vascular cognitive impairment (VCI). Soluble intercellular adhesion molecule-1 (sICAM-1) is upregulated in endothelial dysfunction and promotes an inflammatory response; however, the relationship between sICAM-1 and VCI remains equivocal. Objective: To determine whether sICAM-1 contributes to the prediction of VCI. Methods: Community-dwelling older adults (n=172) from the “Cohort of Obesity, Sarcopenia and Frailty of Older Mexican Adults” (COSFOMA) study were identified as VCI or controls using standard neuropsychological evaluations and neuroimaging. sICAM-1 was quantified using ELISA, and multivariate logistic regression determined the association between sICAM-1 and VCI. Results: 31 VCI cases were identified. sICAM-1 was higher in VCI [VCI: 450.7 (241.6) ng/ml vs. Control: 296.9 (140.9) ng/ml]. sICAM-1 concentrations above the 90th percentile (464.1 ng/mL) was associated with VCI group membership in all models [OR = 6.9 (95% CI: 1.1- 42.2)]. The final saturated model explained 64% of the variance in VCI group membership. Conclusion: High concentrations of sICAM-1 are independently associated with VCI group membership. Efforts to further characterize the relationship between indices of endothelial dysfunction and pathological changes to the aging brain should be further pursued.

